# *β*-Caryophyllene Inhibits Monoacylglycerol Lipase Activity and Increases 2-Arachidonoyl Glycerol Levels In Vivo: A New Mechanism of Endocannabinoid-Mediated Analgesia?[Fn fn5]

**DOI:** 10.1124/molpharm.123.000668

**Published:** 2024-02

**Authors:** Jost Klawitter, Wiebke Weissenborn, Iuliia Gvon, Mackenzie Walz, Jelena Klawitter, Matthew Jackson, Cristina Sempio, Sonja L. Joksimovic, Touraj Shokati, Ingo Just, Uwe Christians, Slobodan M. Todorovic

**Affiliations:** Departments of Anesthesiology (J.K., W.W., I.G., M.W., J.K., M.J., C.S., S.L.J., T.S., U.C., S.M.T.) and Psychiatry (J.K.), School of Medicine, University of Colorado Anschutz Medical Campus, Aurora, Colorado; Department of Pharmacology and Toxicology, Medizinische Hochschule Hannover, Hannover, Germany (W.W., I.G., I.J., U.C.); and Neuroscience Graduate Program, School of Medicine, University of Colorado Denver, Anschutz Medical Campus, Aurora, Colorado (S.M.T.)

## Abstract

The mechanisms of *β*-caryophyllene (BCP)-induced analgesia are not well studied. Here, we tested the efficacy of BCP in an acute postsurgical pain model and evaluated its effect on the endocannabinoid system. Rats were treated with vehicle and 10, 25, 50, and 75 mg/kg BCP. Paw withdrawal responses to mechanical stimuli were evaluated using an electronic von Frey anesthesiometer. Endocannabinoids, including 2-arachidonoylglycerol (2-AG), were also evaluated in plasma and tissues using high-performance liquid chromatography-tandem mass spectrometry. Monoacylglycerol lipase (MAGL) activity was evaluated in vitro as well as ex vivo. We observed a dose-dependent and time-dependent alleviation of hyperalgesia in incised paws up to 85% of the baseline value at 30 minutes after administration of BCP. We also observed dose-dependent increases in the 2-AG levels of about threefold after administration of BCP as compared with vehicle controls. Incubations of spinal cord tissue homogenates from BCP-treated rats with isotope-labeled 2-arachidonoylglycerol-d8 revealed a reduced formation of the isotope-labeled MAGL product 2-AG-d8 as compared with vehicle controls, indicating MAGL enzyme inhibition. In vitro MAGL enzyme activity assessment using 2-AG as the substrate revealed an IC_50_ of 15.8 µM for MAGL inhibition using BCP. These data showed that BCP inhibits MAGL activity in vitro and in vivo, causing 2-AG levels to rise. Since the endocannabinoid 2-AG is a CB1 and CB2 receptor agonist, we propose that 2-AG–mediated cannabinoid receptor activation contributes to BCP’s mechanism of analgesia.

**SIGNIFICANCE STATEMENT:**

*β*-Caryophyllene (BCP) consumption is relatively safe and is approved by the Food and Drug Administration as a flavoring agent, which can be used in cosmetic and food additives. BCP is a potent anti-inflammatory agent that showed substantial antihyperalgesic properties in this study of acute pain suggesting that BCP might be an alternative to opioids. This study shows an additive mechanism (monoacylglycerol lipase inhibition) by which BCP might indirectly alter CB1 and CB2 receptor activity and exhibit its pharmacological properties.

## Introduction

Pain is a pervasive problem that affects millions of Americans each year. Over recent years, the prescription of pain medications, including opioids, has skyrocketed (Trecki, 2019). However, there has been very little progress or innovation in the field of chronic pain (Borsook, 2012). In the past 50 years, the following classes of drugs have been the mainstay of pain treatment: opioids, nonsteroidal anti-inflammatory drugs, antidepressants (amitriptyline), antiepileptics (gabapentin), ziconotide, and ketamine. However, fewer than 10 medications with new mechanisms of action have become available, and only one now in clinical use was designed based on specific mechanisms of action (triptans for migraine) (Borsook, 2012).

*β*-Caryophyllene (BCP; trans–*β*-caryophyllene) is a major volatile plant compound found in large amounts in the essential oils of many different spices and food plants, especially clove oil, essential oils of *Cannabis sativa*, rosemary, and hops (Ghelardini et al., 2001). The Research Institute for Fragrance Materials evaluated BCP’s safety, and the molecule has been approved by the United States Food and Drug Administration and by the European Food Safety Authority as a flavoring agent, which can be used in cosmetic and food additives (Schmitt et al., 2016; Maffei, 2020). BCP has been reported to be a promising agent for treating several disorders, with particular reference to cancer, chronic pain, and inflammation (Francomano et al., 2019; Maffei, 2020). The relatively well established efficacy of BCP in suppressing some forms of pain and inflammation and its low toxicity make it an excellent drug candidate for the treatment of pain. However, its mechanism of analgesia remains largely unknown (Machado et al., 2018). Direct interaction of BCP with the cannabinoid (CB) receptor 2 was previously postulated (Gertsch et al., 2008). Hence, based on its possible binding to the CB2 receptor, BCP was often described as a ligand for this cannabinoid receptor. However, this mechanism and resulting classification were disputed by more recent studies, which observed weak or no direct BCP binding to CB receptors (Santiago et al., 2019; Finlay et al., 2020; Heblinski et al., 2020).

In the present study, we investigated the analgesic properties of BCP on surgically induced hyperalgesia using plantar incision of the hind paw in rats (Brennan et al., 1996; Joksimovic et al., 2019), which is a rodent model considered to be similar in context to the underlying mechanisms and the development of human postoperative pain (Pogatzki-Zahn et al., 2017). Here, we show an alternative mechanism by which BCP may indirectly alter the CB receptor activity. We demonstrate that BCP inhibits monoacylglycerol lipase activity (MAGL) at pharmacologically relevant concentrations and consequently increases the endogenous levels of 2-arachidonoylglycerol (2-AG), an agonist for both CB2 and CB1 receptors. Based on these results, we postulate that increases in levels of the endocannabinoid 2-AG and consequent activation of CB2 and CB1 receptors contribute to the antihyperalgesic and anti-inflammatory properties of BCP.

## Materials and Methods

### Chemicals and Reagents

Solvents and reagents [high-performance liquid chromatography (HPLC)-grade acetonitrile, methanol, water, and the mobile phase constituent, formic acid] used for mobile phases and sample extraction in this study were purchased from Fisher Scientific (Fair Lawn, NJ) and used without further purification. The solvent (ethanol) used for the preparation of stock solutions and PBS was purchased from Sigma-Aldrich Chemicals (St. Louis, MO). All study compound reference materials, including anandamide (arachidonoyl ethanolamide; AEA), 2-AG, 1-arachionoylglycerol (1-AG), docosatetraenoyl ethanolamide (DEA), dihomo-*γ*-linoleoylethanolamide (DH-g-LEA), N-arachidonoyl dopamine (NADA), 2-arachidonoylglycerol ether (2-AGE), O-arachidonoylethanolamide (O-AEA), N-oleoyl dopamine (ODA), oleamide (OLA), linoleoyl ethanolamide (LEA), oleoyl ethanolamine (OEA), palmitoyl ethanolamide (PEA), and stearoyl ethanolamide (SEA), were received from Cayman Chemical Company (Ann Arbor, MI). All isotope-labeled internal standards, including 2-arachidonoylglycerol-d5 (2-AG-d5), anandamide-d4 (AEA-d4), N-arachidonoyl dopamine-d8 (NADA-d8), linoleoyl ethanolamide-d4 (LEA-d4), oleoyl ethanolamide-d4 (OEA-d4), palmitoyl ethanolamide-d4 (PEA-d4), and stearoyl ethanolamide-d3 (SEA-d3), were also from Cayman Chemical Company (Ann Arbor, MI).

For the enrichment of quality control samples, blank human K_2_EDTA plasma from Bioreclamation IVT (Westbury, NY) and charcoal-stripped plasma from BioCheMed (Winchester, VA) were used for the preparation of calibrators.

### Animals

All in vivo experiments were performed with adult female Sprague-Dawley rats (3–5 months old, Envigo, Indianapolis, IN). Animals were housed two per cage and maintained on a 12-hour light/dark cycle with access to food and water ad libitum. Experimental protocols were in accordance with the Guide for the Care and Use of Laboratory Animals (Institute of Laboratory Animal Resources) and were approved by the Animal Care and Use Committee of the University of Colorado Anschutz Medical Campus (Aurora, CO). We used female rats in our study for two main reasons: 1) females are considered to be more prone to pain states when compared with males in both clinical and preclinical studies (Mogil and Chanda, 2005), and 2) the majority of current pain studies in animals are performed using males (Mogil and Chanda, 2005). Thus, we believe that the research focus should also be on female pain perception (Joksimovic et al., 2019; Tat et al., 2020).

### Incision Pain Model

To study the antihyperalgesic effect of BCP after surgery, we used a skin and muscle incision for the induction of postsurgical pain. Our standard model for the induction of postsurgical pain using the skin and muscle incision was described previously (Joksimovic et al., 2018, 2019, 2020; Tat et al., 2020) as follows: animals were anesthetized with isoflurane (2.5% for induction and for maintenance), and the plantar surface of the right paw was incised longitudinally with a blade No. 11. The underlying plantaris muscle was elevated and also longitudinally incised, whereafter the skin was sutured with 5-0 nylon suture with an FS-2 needle. Animals were allowed to recover in cages. All experiments were initiated as early as 2 hours postincision.

### Drug Administration

BCP was dissolved in a solution of ethanol/Cremophor/0.9% saline 1:1:18 in accordance with previous published protocols (Klauke et al., 2014; Yokubaitis et al., 2021). The tested doses of 10 mg/kg, 25 mg/kg, 50 mg/kg, and 75 mg/kg were injected intraperitoneally. Animals were randomly assigned to the dose and vehicle groups, and intraperitoneal injection solutions were prepared by an independent scientist. The operator performing the von Frey testing was blinded to the treatment. The dissolved drug or vehicle solution was administered intraperitoneally at the volume of 2 mL/kg (10 and 25 mg/kg group and eight vehicle controls), 4 mL/kg (50 mg/kg group and four vehicle controls), and 6 mL/kg (75 mg/kg group and four vehicle controls). No volume administration-baseddifferences were observed within the vehicle group. Eight animals (*n* = 8) were in each dose group, and 16 animals were in the vehicle control groups.

### Mechanical Sensitivity

Our standard method for assessing mechanical sensitivity was described previously (Joksimovic et al., 2020; Tat et al., 2020). Briefly: we used the electronic von Frey apparatus (Ugo Basile, Varese, Italy), which utilizes a single rigid filament that exerts pressure to the plantar surface of the paw in a range from 0 to 50 g. Animals were placed in plastic enclosures on a wire mesh stand to habituate for 15 minutes. After habituation, a probe was applied to the plantar surface of the paw through the mesh floor of the stand, and constant force was applied to the midplantar area of the paw. As soon as the exerted pressure of the punctate stimulus reaches the maximum force that the animal can endure, immediate brisk paw withdrawal is noticeable, and the force in grams is displayed by the apparatus, representing a threshold for paw withdrawal response (PWR). Each paw was tested three times, and the average value of threshold PWRs was used in further analysis. Any other voluntary movement of the animal was not considered as a response.

### Sample Collection for Biomarker Assessment

After completion of the mechanical sensitivity testing, animals received a second dose of BCP or vehicle solution. Thirty minutes after this dose, the animals were anesthetized using isoflurane, and blood was drawn; spinal cord and paw tissue surrounding the incision site were collected.

### Endocannabinoid Analysis

Analysis of 14 endocannabinoids and congeners was performed in plasma, paw tissue, and spinal cord tissue. A modification of a previously published assay was used (Sempio et al., 2021). The steps performed for the extraction of plasma and tissue are listed in detail in Supplemental Materials.

### Monoacylglycerol Lipase Activity Assay

A modification of a commercially available MAGL inhibitor screening kit (Item No. 705192, Cayman Chemical Company, Ann Arbor, MI) was used. Initial experiments showed interference of BCP with the colorimetric readout of the assay. Thus, the artificial substrate 4-nitrophenylacetate was substituted with the natural substrate 2-arachidonoylglycerol. Consequently, the assay readout was arachidonic acid (AA) instead of 4-nitrophenol, which was measured using an HPLC with tandem mass spectrometry (MS/MS) approach instead of a colorimetric readout for our experiments in vitro and in vivo (also see Supplemental Fig. 1). A similar methodology has been applied by others (King et al., 2007; Jung and Piomelli, 2016), mostly utilizing 2-oleoylglycerol (2-OG) as the substrate.

### In Vitro MAGL Activity

After addition of 10 µL MAGL (1.25 µg/mL), 10 µL of ethanol solution or BCP solution in ethanol, and 10 µL of 2-AG (0.4 mM) to 150 µL of the MAGL assay buffer [10 mM Tris(hydroxymethyl)aminomethane hydrochloride (Tris-HCl), pH 7.2, 1 mM EDTA] supplied by the kit, the samples were incubated at room temperature for 10 minutes. The total volume of the reaction mixture was 180 µL. The reaction was terminated by the addition of 800 µL methanol including 0.125 µg/mL of the internal standard arachidonic acid-d8 (Cayman Chemical Company, Ann Arbor, MI). Samples were centrifuged, and supernatants were placed into HPLC vials for analysis. The HPLC-MS/MS parameters are listed in Supplemental Materials. Since BCP is very lipophilic and volatile, BCP levels in the assay buffer solution at the end of the incubation were determined (see HPLC-MS/MS analysis of BCP below and in Supplemental Materials).

### In Vivo MAGL Activity

For in vivo MAGL activity assessment, 25–41 mg of spinal cord tissue was homogenized over liquid nitrogen using precooled mortar and pestle. The tissue was added to 85 µL of MAGL assay buffer in a tube on a scale, and the added weight of tissue was recorded. Five microliters of 2-AG-d8 (0.85 mM) were added to the slurry and incubated for 60 minutes at 37°C. The reaction was terminated by the addition of 400 µL methanol including 1.25 µg/mL of the internal standard arachidonic acid-d5 (Cayman Chemical Company, Ann Arbor, MI). Samples were centrifuged, and supernatants were placed into HPLC vials for analysis. The HPLC-MS/MS parameters are listed in Supplemental Materials.

### BCP Analysis

Stock solutions of BCP were prepared in ethanol at 1 mg/mL. This was used to generate calibration curves in the range between 0.0025 µg/mL to 10 µg/mL (0, 0.0025, 0.005, 0.01, 0.02, 0.05, 0.1, 0.4, 1, 4, 10 µg/mL). For tissue analysis, paw tissue and spinal cord tissue were manually homogenized over liquid nitrogen using a precooled mortar and pestle. Approximately 50 mg of tissue was added to a preweighed microcentrifuge tube containing 500 µL of acetonitrile, and the actual tissue weight was noted. Samples were vortexed thoroughly using a Multitube vortexer (Fisher Scientific, Waltham, MA) and centrifuged at 25,000*g* and 4°C for 10 minutes (MR 23i centrifuge with a fiberlite rotor, Thermo Scientific, Waltham, MA). Thirty microliters of the supernatant was transferred into HPLC vials with conical inserts. To this, 5 µL of the internal standard (40 µg/mL limonene) solution was added and briefly vortexed. For plasma, 50 µL of the sample was placed into a 1.5-mL microcentrifuge tube. Then, 5 µL of the internal standard (200 µg/mL limonene) solution was added. Samples were mixed briefly, and 200 µL of acetonitrile was added to each sample, vortexed thoroughly on a multitube vortexer (Fisher Scientific, Waltham, MA), and centrifuged at 25,000*g* and 4°C for 10 minutes (MR 23i centrifuge with a fiberlite rotor, Thermo Scientific, Waltham, MA). One hundred microliters of the supernatant was transferred into an HPLC vial with an insert. Samples were analyzed using an Agilent 1200 series HPLC (Agilent, Santa Clara, CA) interfaced with an ABSciex API5000 MS/MS system (Sciex, Foster City, CA) via an IonDrive Turbo V Source operated in positive atmospheric pressure chemical ionization mode. Ten microliters of extract were injected onto a Halo C8 analytical column (2.7 µm, 3.0 × 100 mm, MAC-MOD Analytical, Chadds Ford, PA), and a gradient using 0.1% formic acid in water and methanol with 0.1% formic acid (1/1, v/v) was applied to achieve the chromatographic separation of BCP and limonene from endogenous compounds. The tandem mass spectrometry detector was operated in positive multiple reaction monitoring mode. The electron potential was set to -10eV and the collision cell exit potential to −12eV. The mass spectrometer was set to acquire BCP and the internal standard limonene (m/z, parent > daughter ion): *m/z =* 205 > 149 (quantifier), *m/z =* 205 > 95 and *m/z =* 205 > 55 (qualifiers) for BCP; *m/z =* 137 > 81 for the internal standard limonene.

### Data Analysis

Once the data were acquired, the MultiQuant, OS-MQ version 1.7 software (Sciex) was used for initial data processing and plasma level determination of lipids and endocannabinoids. Calibration curves were prepared in surrogate buffer. Both assays were validated as considered fit for purpose following generally applicable biomarker validation guidelines with accuracy and precision acceptance criteria of 80%–120% of the nominal concentrations and <20% coefficient of variance, respectively. The resulting metabolite concentrations were normalized to the tissue weight and used for all subsequent statistical analyses.

### Statistical Data Analysis

For statistical analysis, SPSS software version 27 (IBM, Armonk, NY) was used. Data were analyzed without previous log transformation. Distribution statistics included the calculation of means and S.D. Groups were compared using ANOVA in combination with Tukey’s, Dunnett’s, and Bonferroni’s post hoc tests where applicable.

## Results

**Fig. 1.**
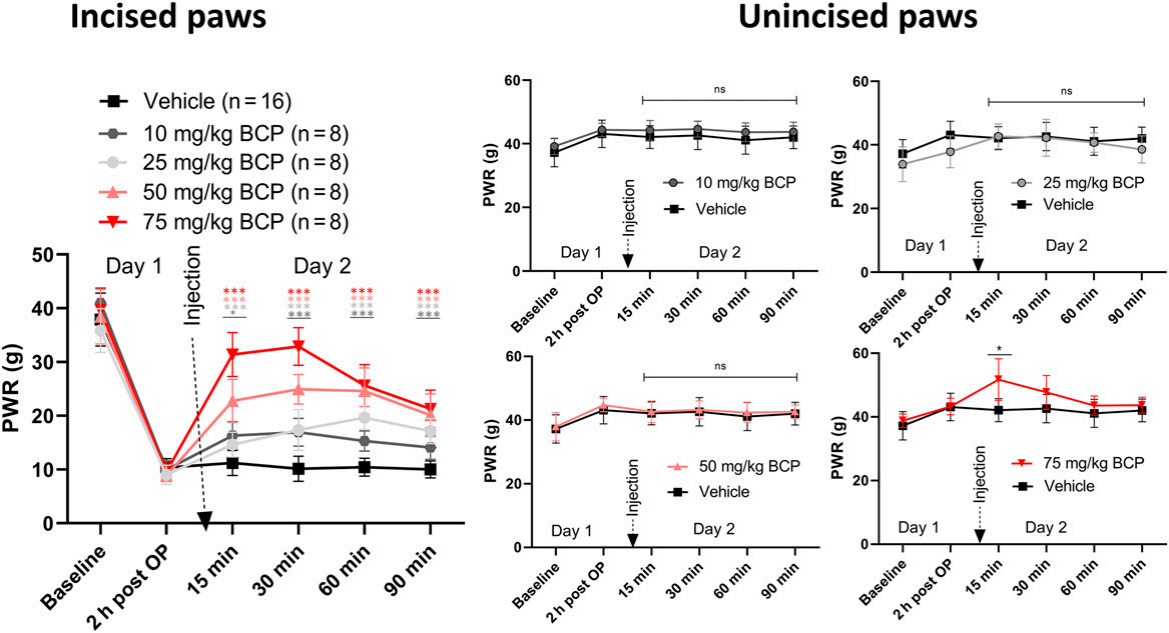
PWR observed using von Frey filament testing. Left side, PWR for the incised right paw for the vehicle control study group (black) and for the study groups with BCP treatment groups at 0 (vehicle), 10, 25, 50, and 75 mg/kg. Right side, the corresponding PWR for the nonincised left paws of the same study groups. Each treatment study group consisted of eight animals (*n* = 8), and the vehicle group consisted of 16 animals (*n* = 16). Data are shown as mean ± S.D. For the incised paw at 15, 30, 60, and 90 minutes after BCP administration, all treatment groups showed higher (*P* < 0.01) mean PWRs compared with the vehicle control group. ANOVA in combination with Dunnett’s post hoc test was performed, and statistically significant changes between the study group and vehicle controls are shown as *P < 0.05; ***P < 0.001. The color coding of the asterisk* corresponds to the study group.

To assess the analgesic properties of BCP and its potential mechanism of action, we focused on the postsurgical incision pain model. The effects of BCP on mechanical hyperanalgesic effects in rats after paw surgery when administered in escalating doses at 10, 25, 50, and 75 mg/kg i.p. were compared with vehicle administration. In animals that underwent skin incision, we focused on mechanical hyperalgesic effects as an important feature of incisional pain for two main reasons: it is very commonly observed in a clinical setting of incisional pain (Zahn and Brennan, 1999; Brennan, 2011; Tat et al., 2020), and, as we published previously, it is sensitive to pharmacological and biochemical modulation (Joksimovic et al., 2020; Tat et al., 2020). The PWRs were determined in the incised right hind paw (Fig. 1, left side) and contralateral nonoperated (left) paws (Fig. 1, right panels). We noticed that BCP caused a prominent dose-dependent increase of the PWRs only in operated paws, indicating a selective antihyperalgesic effect. The baseline PWR for the right paws prior to the incision was 38.5 ± 4.5 g (mean ± S.D.). At 2 hours postsurgery, this value dropped to 9.7 ± 4.5 g, indicating hyperalgesic effects, with a drop of 75% of the preincision value. Whereas the vehicle group (*n* = 16) remained at an average of 10.5 ± 2.0 g during the treatment period, all BCP-treated animals showed diminished paw withdrawal responses at 15 and 30 minutes postinjection. Comparison of the time points 15, 30, 60, and 90 minutes after injection of vehicle or BCP using ANOVA showed *P* values of 2.7·10^−16^, 1.8·10^−18^, 6.8·10^−15^, and 9.5·10^−16^, respectively. The maximal improvement of PWR compared with vehicle treatment (in % PWR of vehicle control) were observed at 30 minutes for all tested doses except for the 25 mg/kg dose, where the maximal effect was slightly higher at 60 minutes as compared with 30 minutes (188% versus 170%). The maximal values (in % PWR of vehicle control) and the 95% confidence intervals (CIs) at these time points were 167% (95% CI, 145%–188%), 188% (95% CI, 170%–206%), 245% (95% CI, 223–268%), and 324% (95% CI, 295%–353%) for the 10, 25, 50, and 75 mg/kg treatment groups, respectively. Post hoc Tukey analysis showed that PWRs in all BCP treatment groups were statistically significantly improved (all *P* < 0.001) compared with vehicle controls at all time points with the exception of 10 mg/kg after 15 minutes (Fig. 1). The changes were most pronounced for the 75 mg/kg BCP treatment group (*n* = 8), showing PWR improvements with 31.4 ± 4.1 and 32.9 ± 3.5 g (mean ± S.D.) for 30 and 60 minutes postinjection, respectively. The PWRs for this group were only 15% lower than the presurgery values (38.5 ± 4.5 g). The PWRs were also evaluated in the nonincised left hind paws of study animals (Fig. 1, right side). On the day of the PWR assessments, the left paws showed an average of 41.4 ± 2.1 g (mean ± S.D.) for the vehicle group. Except for a moderate and transient increase in PWR in the 75 mg/kg group at 15 minutes post i.p. injection (51.7 ± 6.6 g), there were no changes in the left paw PWRs among all treatment groups compared with the vehicle control. This indicates that BCP selectively alleviated mechanical hyperalgesia in affected paws following surgical skin incision.

**Fig. 2.**
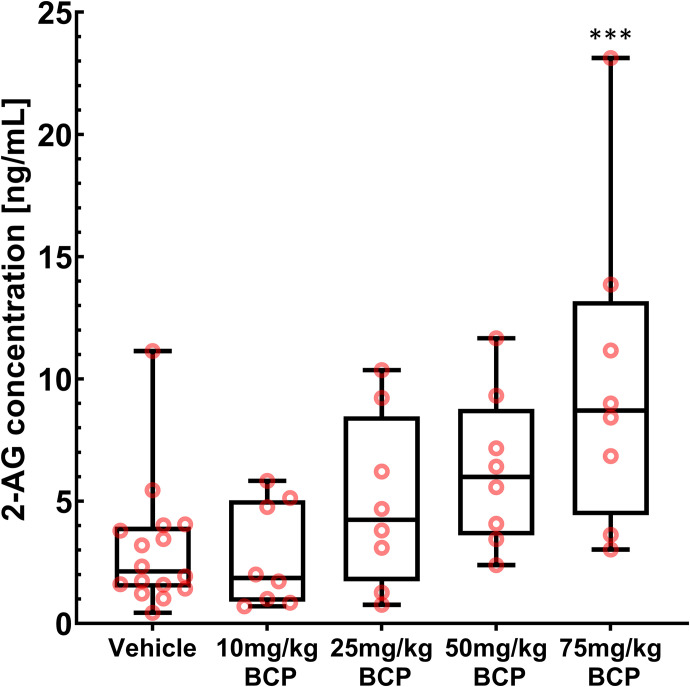
Determination of 2-AG plasma levels after treatment with vehicle solution or BCP in the postsurgical pain model. 2-AG levels were determined as part of an HPLC-MS/MS–based endocannabinoid multiplex assay. Each BCP treatment study group consisted of eight animals (*n* = 8), and the vehicle group consisted of 16 animals (*n* = 16). The black line inside each box marks the median of the corresponding 2-AG concentration distribution. The lower box boundaries mark the 25th and 75th percentiles of each 2-AG concentration distribution, respectively. Whiskers that appear above and below the box boundaries show the largest and smallest observed concentrations. ANOVA in combination with Tukey’s post hoc test was performed, and statistically significant changes between study groups are shown as *** *P* < 0.001.

Next, to further gain insight into the mechanism of BCP-induced antihyperalgesia in this pain model, we evaluated changes in endocannabinoid plasma and tissue concentrations using a comprehensive endocannabinoid analysis including 14 endocannabinoids and congeners in plasma and tissue samples. We noticed that plasma 2-AG concentrations increased in parallel with increasing BCP doses (Fig. 2). Of the 14 endocannabinoids included in the assay, only eight had quantifiable concentrations, and only 2-AG showed any obvious differences (change larger than factor 3). Hence, the statistical analysis focused on 2-AG only. Mean 2-AG plasma levels for the vehicle control group were 3.0 ± 2.6 ng/mL (mean ± S.D.). Except for the 10 mg/kg BCP treatment group, all other treatment groups showed increased 2-AG plasma levels, with the highest plasma concentration for the 75-mg/kg dose group, with 9.9 ± 6.4 ng/mL (330% of vehicle control) (ANOVA, *P* = 0.00086, Tukey post hoc test, all *P* < 0.001). Absolute tissue concentrations of 2-AG in paw tissue and spinal cord tissue resulted in no relevant changes between study groups. However, we found a statistically significant (*P* = 0.0024) correlation between the concentrations of 2-AG and BCP in the spinal cord tissue (Pearson correlation coefficient r = 0.518). A linear regression analysis is shown in Supplemental Fig. 2 (R^2^ = 0.268).

**Fig. 3.**
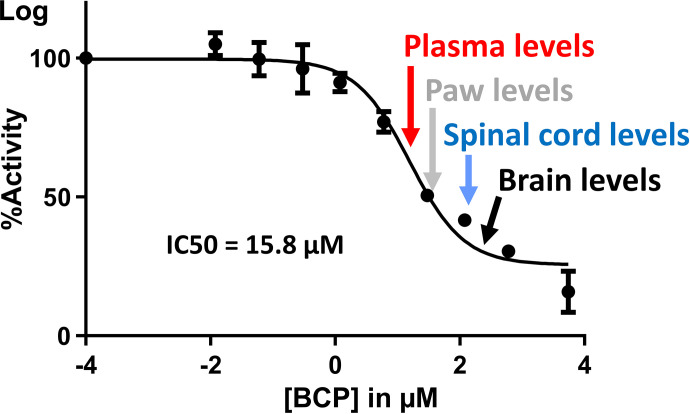
Determination of MAGL inhibition in vitro using a modification of a commercially available MAGL inhibitor screening kit. The artificial substrate of the kit was replaced with the natural MAGL substrate 2-AG. MAGL inhibition by BCP was determined based on the natural substrate 2-AG, which is hydrolyzed by MAGL to glycerol and AA. AA concentrations were determined using HPLC-MS/MS. Determination of the enzyme inhibition was performed on 3 consecutive days, with two determinations per BCP level on each day (total *n* = 6). The in vitro enzyme inhibition data are shown as mean ± S.D., and enzyme inhibition was plotted using GraphPad Prism software (version 9.4.1, GraphPad, San Diego, CA). Please note that some of the error bars are hidden behind the data point symbols.

To test our hypothesis that the increase of 2-AG concentrations was caused by an effect of BCP on MAGL activity, a commercially available MAGL inhibitor screening assay kit (Cayman Chemicals) was initially tested. For this application, the assay showed interference of BCP with the colorimetric readout. Thus, a modification of the assay was developed in which the natural MAGL substrate 2-AG was used, and the product, AA, was quantified using HPLC-MS/MS (see Supplemental Fig. 1). After the development and validation of the assay (see *Methods*), it was applied on 3 consecutive days, with three assessments per day (Fig. 3). Under these conditions, the IC_50_ for BCP was determined to be 15.8 µM (95% CI, 12.0–20.9 and a coefficient of determination for the curve fit of R^2^ = 0.962).

**Fig. 4.**
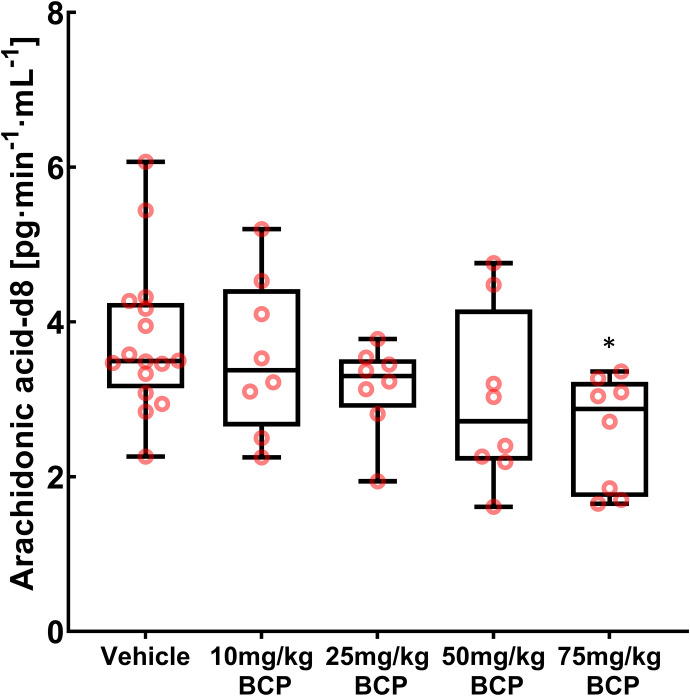
Ex vivo determination of MAGL activity in spinal cord tissue. MAGL activity evaluation was performed using the deuterated substrate 2-AG-d8, which is hydrolyzed by MAGL to glycerol and arachidonic acid-d8 (AA-d8). AA-d8 concentrations were determined using HPLC-MS/MS using AA-d5 as internal standard. Each BCP treatment study group consisted of eight animals (*n* = 8), and the vehicle group consisted of 16 animals (*n* = 16). The black line inside each box marks the median of the corresponding AA-d8 formation distribution. The lower box boundaries mark the 25th and 75th percentiles of each AA-d8 formation distribution, respectively. Whiskers that appear above and below the box boundaries show the largest and smallest observed AA-d8 formation rates. ANOVA in combination with Tukey’s post hoc test was performed, and statistically significant changes between study groups are shown as * *P* < 0.05.

Monoacylglycerol lipase activity was also assessed in spinal cord tissues. Animals were treated with BCP at 0 mg/kg (vehicle controls) as well as 10, 25, 50, and 75 mg/kg. Tissue was harvested 30 minutes after BCP administration, frozen in liquid nitrogen, and stored at −70°C or below until analysis. Due to the natural abundance of 2-AG and arachidonic acid in tissue, homogenized samples were incubated in a slurry with MAGL activity assay buffer and labeled 2-AG-d8. After the incubation period, the tissue was extracted, and the AA-d8 content was analyzed (for details, see *Methods* and Supplemental Fig. 1). Formation of AA-d8 was reduced in spinal cord tissues of animals previously treated with BCP (Fig. 4). Vehicle controls showed an AA-d8 formation rate of 3.8 ± 1.0 pg·mg^−1^·min^−1^ (mean ± S.D.). The values for the treatment groups were 3.6 ± 1.0 pg·mg^−1^·min^−1^, 3.2 ± 0.6 pg·mg^−1^·min^−1^, 3.0 ± 1.1, and 2.6 ± 0.7 (mean ± S.D.) for the 10, 25, 50, and 75 mg/kg treatment groups, respectively. The ANOVA test showed statistically significant differences with *P* = 0.043 among study groups. Tukey’s post hoc test showed a changed AA-d8 formation rate in the 75-mg/kg treatment group (*P* = 0.036) as compared with the vehicle control group. The change was a reduction of AA-d8 formation rate by 31% (95% CI, 11%–54%).

**Fig. 5.**
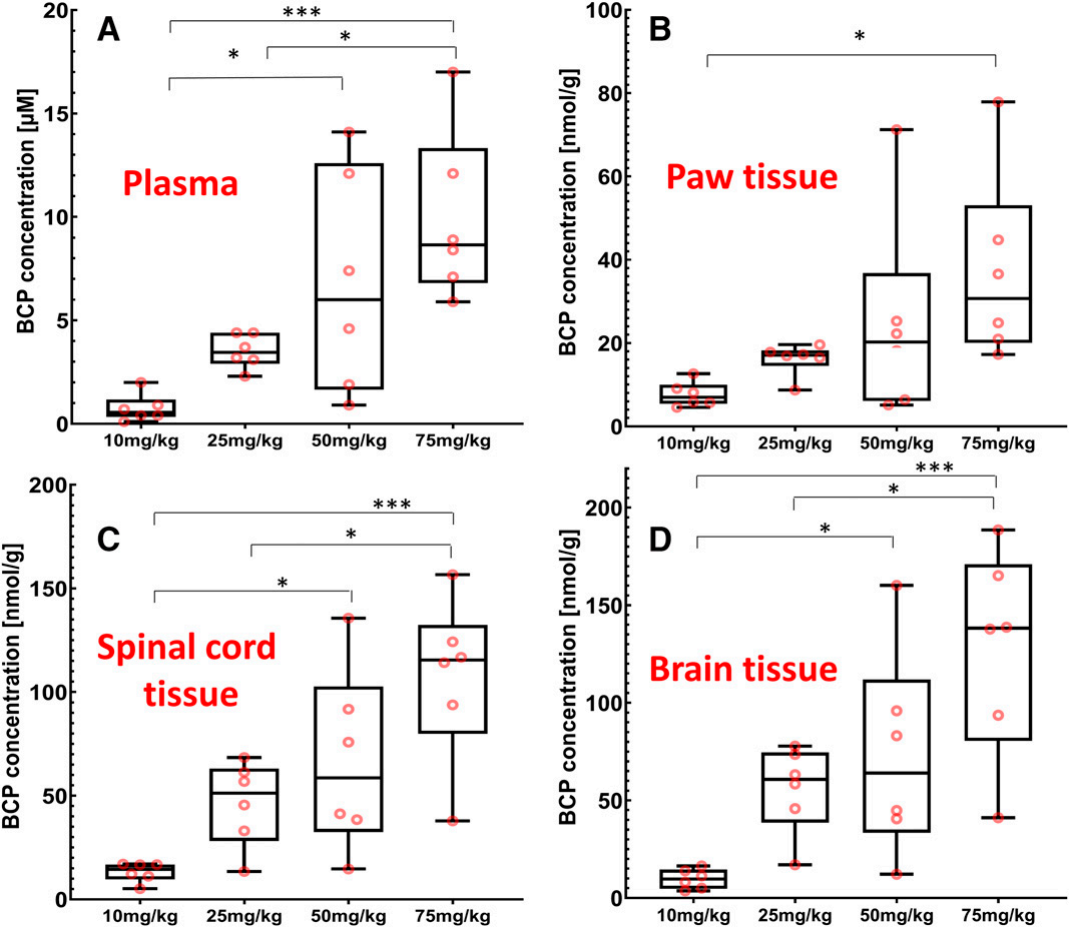
Boxplot of BCP concentrations in plasma and tissue 30 minutes after BCP administration. To determine the plasma and tissue concentrations, 24 animals were assigned to five study groups (vehicle, 10, 25, 50, and 75 mg/kg of BCP). All data are *n* = 6 animals per treatment group. The black line inside each box marks the median of the corresponding concentration distribution. The lower box boundaries mark the 25th and 75th percentiles of each concentration distribution, respectively. Whiskers that appear above and below the box boundaries show the largest and smallest observed concentrations. Concentrations in plasma (A), paw tissue (B), spinal cord (C), and brain tissue (D) are shown. ANOVA in combination with Tukey’s post hoc test was performed, and statistically significant changes between study groups are shown as *P < 0.05; ***P < 0.001.

Next, we determined BCP drug levels in plasma, spinal cord, paw, and brain tissue 30 minutes after administration of a single BCP intraperitoneal dose to estimate plasma and tissue concentrations and to correlate in vitro data to in vivo concentrations at the time when the largest antihyperalgesic effect was observed. An HPLC–atmospheric pressure chemical ionization multiple reaction monitoring MS/MS assay was developed to quantify BCP with high specificity and selectivity. Representative extracted ion chromatograms of BCP in plasma samples are shown in Supplemental Fig. 3. The assay was linear from 0.49 to 490 nmol. All plasma and tissue samples of treated animals were within the linear range of the assay. Tissue levels were normalized to the amount of tissue extracted with acetonitrile and are presented as nmol/g of tissue to allow for easy comparison with plasma levels, which are presented in nmol/mL (see Fig. 5, A–C). BCP plasma levels were 0.75 ± 0.67, 3.5 ± 0.8, 6.8 ± 5.4, and 9.9 ± 4.1 nmol/mL (mean ± S.D.) for the 10-, 25-, 50-, and 75-mg/kg treatment groups, respectively. Paw tissue levels were higher than plasma levels, with 7.7 ± 3.0, 16.1 ± 3.8, 24.8 ± 24.2, and 37.1 ± 22.5 nmol/g (mean ± S.D.) for the 10-, 25-, 50-, and 75-mg/kg treatment groups, respectively. Spinal cord BCP concentrations reached 13.2 ± 4.7, 46.4 ± 20.0, 66.3 ± 43.4, and 107.2 ± 39.7 nmol/g (mean ± S.D.) for the 10-, 25-, 50-, and 75-mg/kg treatment groups, respectively. Interestingly, the brain concentrations were 12-fold higher in comparison with plasma and up to threefold higher in comparison with paw tissue depending on the treatment group. Brain BCP concentrations reached 9.8 ± 5.0, 55.1 ± 22.0, 72.8 ± 52.8, and 127.5 ± 52.9 nmol/g (mean ± S.D.) for the 10-, 25-, 50-, and 75-mg/kg treatment groups, respectively (Fig. 5, A–D). Comparison of the BCP concentrations among the different dose groups using ANOVAs showed differences with *P* = 0.0010, *P* = 0.0379, *P* = 0.0004, and *P* = 0.0005 for plasma, paw tissue, spinal cord tissue, and brain tissue, respectively.

## Discussion

In this study, we showed that BCP inhibited MAGL activity at pharmacologically relevant concentrations and consequently increased the levels of the endogenous CB receptor agonist 2-AG. Hence, we posit that the inhibition of MAGL may contribute/add to the putative CB receptor agonism of BCP. However, several studies had questioned if BCP acts as a direct CB2 receptor agonist (Santiago et al., 2019; Finlay et al., 2020; Heblinski et al., 2020). Thus, it was important to investigate if BCP may indirectly act on the CB2 receptor via 2-AG.

BCP is approved by the United States Food and Drug Administration and European agencies as a food additive, taste enhancer, and flavoring agent (Chicca et al., 2014). It is also reported to display important pharmacological activities, such as anticancer, cardioprotective, hepatoprotective, gastroprotective, antioxidant, anti‐inflammatory, antimicrobial, immune‐modulatory, and neuroprotective effects (Chicca et al., 2014; Fidyt et al., 2016; Sharma et al., 2016; Machado et al., 2018). Some studies have also proposed that BCP may be efficacious for the treatment of several neurologic diseases and disorders, such as cerebral ischemia, brain lesions, neuro‐inflammation, and problems in cortical, hippocampal, and cerebellar neurons and glial cells (Zhang et al., 2008, 2009; Pertwee, 2009; Lanciego et al., 2011; Zarruk et al., 2012; Machado et al., 2018). The analgesic effect of BCP in essential oils and other formulations has been previously studied (Menezes et al., 2007; Sousa et al., 2008; Mishra et al., 2010; Klauke et al., 2014; Khalilzadeh et al., 2015; Segat et al., 2017; Hernandez-Leon et al., 2020). Indeed, BCP-rich essential oils have demonstrated antinociceptive effects at 25–62.5 mg/kg (Khalilzadeh et al., 2015), 50–75 mg/kg (Mishra et al., 2010), and 100–400 mg/kg (Menezes et al., 2007; Sousa et al., 2008) (per os). However, BCP content in essential oils is often variable, and surprisingly, pure BCP displays similar analgesic activities as several essential oils in which BCP is a major active compound (Fidyt et al., 2016). This might be related to bioavailability and contribution of other essential oil components to the effect of BCP. Pure BCP preparations were tested at various concentrations in single and multiple administration dose designs. In this context, BCP showed to be antinociceptive at 1–5 mg/kg in mice (Klauke et al., 2014), 2.6 mg/kg per day for 2 weeks in mice (Fiorenzani et al., 2014) and 25 mg/kg (Segat et al., 2017). Most of these models tested the antinociceptive effect of BCP in formalin, acetic acid, and tail immersion tests. To our knowledge, the antihyperalgesic effects of BCP in an acute postsurgical pain model have not previously been studied. Here, we observed a BCP dose-dependent increase of the PWR preferentially in surgically injured paws using a clinically relevant model of postoperative pain (Fig. 1). However, we cannot exclude an additional anti-inflammatory effect contributing to the analgesic effect. The lowest BCP dose tested (10 mg/kg) already showed substantially higher PWRs (145%–167%) at 15, 30, 60, and 90 minutes after injection compared with vehicle controls (100%). Administration of 75 mg/kg resulted in paw withdrawal responses at 30 minutes postinjection (32.9 ± 3.5 g) that were 85% of the mean PWR for all baseline values prior to the incision (38.5 ± 4.5 g), indicating a potent antihyperalgesic effect of BCP. Our data demonstrated a remarkably selective postoperative pain relief in incised paws, whereas nonoperated paws were only slightly affected with the highest dose of BCP. The difference to the effective doses in the mice formalin/acetic acid and tail immersion tests is likely based on the characteristics of the different pain models and/or different species tested.

Key components of the endocannabinoid system are expressed throughout nociceptive pathways: in the periphery on primary afferent neurons, in the dorsal horn of the spinal cord, and in multiple supraspinal regions of the brain associated with pain perception and modulation (Finn et al., 2021). As a result, targeting the endocannabinoid system via enhancement of the levels of endogenous cannabinoids [e.g., with fatty acid amide hydrolase (FAAH) or MAGL inhibitors] or exogenous cannabinoid ligands (e.g., CB1 or CB2 receptor agonists) can reduce nociceptive transmission at all three of these neurophysiological levels (Finn et al., 2021). Here, we evaluated the effects of BCP treatment on the endocannabinoid system and observed changes in the circulating endocannabinoid 2-AG (Fig. 2). These results led us to hypothesize that BCP increases 2-AG levels via inhibition of MAGL.

We determined that the IC_50_ for BCP-mediated inhibition of MAGL activity in vitro was 15.8 µM. At the highest BCP concentration used, the lowest MAGL activity was observed with only 15.8 ± 3.0% residual activity. There are two possible explanations for the residual MAGL activity in this in vitro assay. One is that BCP is very volatile and lipophilic. The volume of organic solvent in this assay is limited. Thus, creating a high concentration standard for enriching the incubation vessel is limited by the lipophilic nature of BCP. Higher BCP levels, which could not be tested for the aforementioned reason, might result in more complete inhibition of MAGL activity. However, without any physiologic matrix, just the assay buffer, this could not be tested. Another possible reason could be competition with the substrate. Substrate concentrations in the in vitro assay were 22 µM in the incubation vessel. These substrate concentrations were chosen based on substrate linearity experiments (data not shown). For all inhibition experiments to define an IC_50_, a high, constant concentration of substrate should be present so that the enzyme can react at an appreciable rate. The substrate (2-AG) concentrations used in vitro surpassed those observed by us in plasma and by others in the brain (Buczynski and Parsons, 2010).

To further test MAGL activity in a more physiologic environment, a similar approach was applied to an ex vivo study. MAGL activity was assessed ex vivo in tissue homogenates of animals from the BCP treatment groups. For this, deuterated 2-AG (2-AG-d8, Supplemental Fig. 1) was used as the MAGL substrate. After incubation of the tissue slurry, samples were extracted, and the MAGL product AA-d8 was quantified using HPLC-MS/MS. A decrease of AA-d8 formation was observed in spinal cord tissue from 75 mg/kg–treated animals, indicating a decreased hydrolysis of 2-AG-d8 in this treatment group (Fig. 4). This data demonstrated that BCP inhibits MAGL in vitro and in vivo and most likely explains why BCP increased 2-AG levels in plasma and spinal cord tissue (Supplemental Fig. 2) in a dose-dependent manner. It is well established that MAGL inhibition and the resulting increased 2-AG levels have antinociceptive and anti-inflammatory effects (Long et al., 2009; Kinsey et al., 2013; Ignatowska-Jankowska et al., 2015; Sun et al., 2019). Importantly, it was previously reported that 2-AG is an endogenous agonist for both CB1 and CB2 receptors (Baggelaar et al., 2018). Therefore, we posit that the BCP-induced increased levels of 2-AG could contribute to the antihyperalgesic effects or BCP that we observed in incised paws. Although an IC_50_ determined in our in vitro MAGL inhibitor assay might not be identical to that of tissue MAGL, our conclusion is supported by the fact that labeled 2-AG incubations of tissues extracted from BCP-treated rats also confirmed MAGL inhibition. Based on this data, an inhibition (partial and possibly competitive) of MAGL activity by BCP was observed at the concentrations used in this study.

We used an HPLC-MS/MS approach to determine if concentrations of BCP that are required for MAGL inhibition can be reached in vivo. Indeed, our data showed that BCP concentrations in both plasma and examined tissues in vivo were severalfold higher than the calculated IC_50_ for MAGL inhibition in vitro. Due to the different routes of administration and formulations of BCP, which is a lipophilic and volatile terpene, it is essential to determine plasma and tissue levels to accurately interpret in vitro and in vivo experiments as well as allowing for a comparison of data gained from this study to other studies. Plasma BCP levels 30 minutes after a single administration of BCP in nonincised animals did show a concentration-dependent increase among the study groups (Fig. 5). The increase was linear for the 10-, 25-, 50-, and 75-mg/kg treatment groups, with mean values of 0.74, 3.51, 6.84, and 9.90 nmol/mL (µM), respectively. This linear level increase was reflected in paw and spinal cord tissue levels as well. BCP levels were increased in paw tissue and even further accumulated in spinal cord and brain tissue as compared with plasma, which can be explained by the very lipophilic nature of BCP. The concentrations of BCP observed are generally in alignment with the literature (Liu et al., 2013; Varga et al., 2018). Varga et al. (2018) determined serum levels of 1.2 nmol/mL 30 minutes after single-dose administration of 10 mg/kg BCP i.p. in mice compared with 0.74 nmol/mL observed in rat plasma in the present study 30 minutes after the second BCP dose. Others performed pharmacokinetic analysis after oral administration of 50 mg/kg BCP in rats (Liu et al., 2013; Lou et al., 2017) and determined C_max_ levels 2.5–10 nmol/mL at 1.5–3 hours after administration depending on the vehicle solutions used. In the present study, we observed average plasma levels of 6.8 nmol/mL (µM) in the 50-mg/kg group. It is known that very lipophilic drugs administered by intraperitoneal injection may result in very high central nervous system concentrations due to excellent blood supply in combination with high lipophilicity of the brain/spinal cord. Afterward, redistribution takes place, resulting in lowering of brain/spinal cord concentrations and a secondary increase in plasma concentrations. In this model, concentrations were not yet at a steady state. Thus, direct comparison of the BCP kinetics in different studies using either intraperitoneal injections or oral administration is difficult.

It has previously been proposed that BCP may be a putative CB2 receptor agonist (Gertsch et al., 2008; Irrera et al., 2019, 2020; Mensching et al., 2020; Alberti et al., 2021). Indeed, many characteristic properties of BCP, such as the antihyperalgesic and anti-inflammatory properties, can be explained via this mechanism of action. Although we did not evaluate direct binding/agonism of BCP to the CB receptors, our data strongly suggest that MAGL inhibition, and, consequently, increased levels of 2-AG, may at least contribute to the effective pain relief observed with BCP administration in rats after paw incision. Further studies are needed to evaluate this mechanism of action of BCP in preclinical pain models.

## Abbreviations

2-AG: 2-arachidonoylglycerol
AA: arachidonic acid
BCP: *β*-caryophyllene
CB: cannabinoid
CI: confidence interval
HPLC: high-performance liquid chromatography
MAGL: monoacylglycerol lipase
MS/MS: tandem mass spectrometry
PWR: paw withdrawal response
